# Lightweight Authentication Mechanism for Industrial IoT Environment Combining Elliptic Curve Cryptography and Trusted Token

**DOI:** 10.3390/s23104970

**Published:** 2023-05-22

**Authors:** Yu-Sheng Yang, Shih-Hsiung Lee, Jie-Min Wang, Chu-Sing Yang, Yuen-Min Huang, Ting-Wei Hou

**Affiliations:** 1 Department of Engineering Science, National Cheng Kung University, Tainan City 701, Taiwan; n98991108@mail.ncku.edu.tw (Y.-S.Y.); huang@mail.ncku.edu.tw (Y.-M.H.); houtw@mail.ncku.edu.tw (T.-W.H.); 2 Department of Intelligent Commerce, National Kaohsiung University of Science and Technology, Kaohsiung City 824, Taiwan; 3 Institute of Computer and Communication Engineering, National Cheng Kung University, Tainan City 701, Taiwan; n26102042@gs.ncku.edu.tw; 4 Miin Wu School of Computing, National Cheng Kung University, Tainan City 701, Taiwan; csyang@ee.ncku.edu.tw

**Keywords:** authentication, elliptic curve cryptography, token, industrial Internet of Things (IIoT)

## Abstract

With the promotion of Industry 4.0, which emphasizes interconnected and intelligent devices, several factories have introduced numerous terminal Internet of Things (IoT) devices to collect relevant data or monitor the health status of equipment. The collected data are transmitted back to the backend server through network transmission by the terminal IoT devices. However, as devices communicate with each other over a network, the entire transmission environment faces significant security issues. When an attacker connects to a factory network, they can easily steal the transmitted data and tamper with them or send false data to the backend server, causing abnormal data in the entire environment. This study focuses on investigating how to ensure that data transmission in a factory environment originates from legitimate devices and that related confidential data are encrypted and packaged. This paper proposes an authentication mechanism between terminal IoT devices and backend servers based on elliptic curve cryptography and trusted tokens with packet encryption using the TLS protocol. Before communication between terminal IoT devices and backend servers can occur, the authentication mechanism proposed in this paper must first be implemented to confirm the identity of the devices and, thus, the problem of attackers imitating terminal IoT devices transmitting false data is resolved. The packets communicated between devices are also encrypted, preventing attackers from knowing their content even if they steal the packets. The authentication mechanism proposed in this paper ensures the source and correctness of the data. In terms of security analysis, the proposed mechanism in this paper effectively withstands replay attacks, eavesdropping attacks, man-in-the-middle attacks, and simulated attacks. Additionally, the mechanism supports mutual authentication and forward secrecy. In the experimental results, the proposed mechanism demonstrates approximately 73% improvement in efficiency through the lightweight characteristics of elliptic curve cryptography. Moreover, in the analysis of time complexity, the proposed mechanism exhibits significant effectiveness.

## 1. Introduction

The industrial Internet of Things (IIoT) [1,2] began in the 1980s when some companies started using automation technologies to increase production efficiency and reduce costs. With the development of network technology, the concept of IIoT gradually matured and received increasing attention in the early 2000s. IIoT is currently divided into three stages. The first stage was in the early days of the Internet of Things (IoT), when the IIoT technology mainly focused on monitoring individual devices, including automation technologies and sensors. From 2000 to 2010, IIoT technology entered the second stage, which involved rapid development. The popularity of cloud computing has enabled the storage and application of industrial data for other related purposes. Additionally, the rapid development of artificial intelligence and machine learning has allowed certain devices to optimize their operations. Furthermore, advancements in wireless communication technology have facilitated the transmission of data collected by terminal IoT devices through wireless networks. However, as the IIoT has rapidly developed and convenience has quickly increased, several security issues have been discovered. Unlike traditional industrial environments, today’s industrial environments use wireless networks as the transmission medium between terminal IoT devices and backend servers. However, such network environments lack information security measures. Once malicious individuals gain access to a network in an industrial environment, they can easily obtain relevant transmission packets within the network and transmit packets to devices in the network.

In today’s industrial environment, most data communications are conducted using the Modbus protocol [3]. Modbus is a popular serial communication protocol owing to its simplicity, ease of use, and scalability, and is used in several industrial environments. However, Modbus has a major drawback: it transmits data in plaintext, which indicates that if a packet is intercepted by malicious actors, they can easily decode its content. This creates significant privacy vulnerabilities during data transmission. In terminal IoT devices, as there is no mutual authentication function between the terminal IoT devices and the backend servers in today’s industrial environment, servers that collect data from the backend will accept data sent from any device. This indicates that if malicious actors gain access to a network in this industrial environment, they can transmit fake data to the backend server, causing data errors, inaccurate assessments of the device’s maintenance status, and unnecessary increases in costs. In addition, using the RSA encryption algorithm to implement the authentication mechanism has been a mainstream approach in the past. The biggest problems with the RSA algorithm are the long encryption and decryption times, requiring significant computational resources. However, most end devices in industrial environments lack sufficient computational resources, which leads to difficulties or excessive processing time for many of these devices.

To address these issues, this study uses the TLSv1.3 protocol [4] to enhance the Modbus protocol in terms of communication. The TLSv1.3 protocol is used to protect network transmission security. It is used to transmit encrypted data to protect the security and privacy of the transmitted data. Through the TLSv1.3 protocol, it can be ensured that the transmitted data will not be exposed owing to theft. Currently, several websites use TLS protocols to enhance their communication security. To authenticate terminal IoT devices and backend servers, this study uses elliptic curve cryptography [5] combined with trusted tokens (JSON web token or JWT) [6] to implement an authentication system between terminal IoT devices and backend servers. This method ensures that the data received by the backend server are from a legitimate device, thereby solving the problem of whether the data source is legal. Several authentication systems have previously used RSA encryption algorithms [7] to implement identity authentication processes. In this study, elliptic curve cryptography was chosen as the implementation method because it can achieve the same level of security as RSA with a shorter key length. Mahto et al. [8] experimentally demonstrated that elliptic curve cryptography is superior to RSA in terms of efficiency and performance and requires fewer computational resources, making it more suitable for resource-limited terminal IoT devices. The contributions of this study are as follows.

1. Implementation of an authentication system based on elliptic curve cryptography and tokens to protect IIoT environments better with improved security.
2. Faster authentication efficiency compared with related authentication systems.

The remainder of this paper is organized as follows: Section 2 summarizes the related studies. In Section 3, the problems that must be addressed are defined. Section 4 introduces the proposed architecture. Section 5 presents the experimental results and a security analysis. Finally, Section 6 presents the conclusions.

## 2. Related Works

This section introduces related works, including those regarding the security and authentication of the IoT.

### 2.1. Security of Industrial Internet of Things

With the development of the IIoT, various information security-related issues have gradually emerged. In this study, different security issues and their solutions in the IIoT are analyzed from three perspectives. Network security aims to protect the entire IIoT from invasion and attacks by implementing security measures, such as setting up firewalls, implementing security protocols, and establishing secure connections. Second, data security aims to ensure that the data transmitted in the IIoT are not invaded or modified, and that data security can be ensured through encryption and decryption. Finally, the security of IIoT devices is crucial to ensure the security of the entire IIoT system. It is necessary to ensure that the devices are not easily invaded or attacked, and that they can protect their internal resources and systems, which can be achieved through authentication and encryption. Boopalan et al. [9] discussed the security of IIoT data and the challenges that arise with federated learning. Sadhu et al. [10] proposed various threats to the security and privacy of the IoT and the corresponding response strategies. Ahanger et al. [11] explored the vulnerabilities of IoT, related attacks, and their impacts, identified weak links, and proposed effective remedial measures and defect-tracking technology. Sengupta et al. [12] discussed various attack modes encountered in the IIoT and proposed corresponding solutions. Job et al. [13] proposed security issues encountered by devices in a multi-industrial environment that already used supervisory control and data acquisition (SCADA) [14] for device monitoring and control, and suggested relevant research areas. Ferrag et al. [15] proposed a network security dataset for IIoT applications called the Edge-IIoTset for machine learning in the IIoT. Atutxa et al. [16] delegated server certificate verification in the DTLS handshake process to resource-rich servers, reducing the overall handshake time by 50–60% and CPU usage on devices with fewer resources. Zhou et al. [17] proposed a distributed DDoS mitigation solution that distributes traffic analysis tasks to multiple locations. Therefore, this paper proposes a lightweight authentication mechanism that combines elliptical curve cryptography and trusted tokens to enhance the security of the IIoT environment.

### 2.2. Authentication in IIoT Environment

IoT technology has become an important area of focus for several businesses today; however, it has also raised several security concerns, such as identity authentication and the protection of data privacy. To address these issues, researchers have begun exploring various key negotiation protocols and identity authentication mechanisms, continuously innovating and improving them. Among these protocols and mechanisms, elliptic curve cryptography is the most widely used. It provides higher security, smaller keys, and faster processing. In addition, hash functions are important tools for implementing secure authentication systems that can transform large amounts of data into shorter hash values while maintaining the uniqueness and irreversibility of the hash value. The paper [18] utilized JWT for identity authentication in zero trust networks (ZTN). Since zero trust networks stipulate that no device in the network infrastructure can be trusted, they cannot guarantee that the holder of a JWT is the expected registrant before registration. Therefore, there are potential security issues when using JWT for one-time token (OTT) registration. The solution is to embed the JWT as encrypted metadata within a non-fungible token (NFT) to ensure ownership of the OTT. The JWT is encrypted using the blockchain public key of the expected registrant, and the blockchain ensures ownership of the JWT by mapping it to the blockchain public address of the intended owner. In another paper [19], an identity authentication mechanism based on elliptic curve cryptography was proposed for SCADA systems, using OPTIGA Trust X as the device, which provides secure storage for ECC keys. Sharma et al. [20] explored device identity authentication using password-based, one-time password (OTP)-based, and certificate-based methods, analyzing the security of these three authentication approaches in the context of the Internet of Things (IoT). Yang et al. [21] discussed the importance of emergency logistics for supply assurance during emergencies and proposed a lightweight certificateless authentication protocol (CL-LAP) without bilinear pairing, which effectively reduces the authentication time and cost. Dammak et al. [22] proposed a token-based authentication protocol (TBLUA) that has low requirements for computing and storage resources and fast verification time. This mechanism enhances the robustness of identity authentication based on token technology and is suitable for use in resource-constrained IoT environments. Ahmed et al. [23] proposed an authentication mechanism based on the JWT that uses a timestamp to record client requests and server response times, resolving disputes over token validity when the client identity is revoked. Nyangaresi [24] proposed an identity authentication mechanism that uses elliptical curve cryptography, fuzzy extractors, and biometric tokens for applications in 5G networks. Das et al. [25] proposed a key negotiation protocol called LACKA-IoT, which uses elliptic curve cryptography and hash functions to implement the authentication system and uses the ROR model for security analysis. Lara et al. [26] proposed an elliptic curve cryptography-based authentication mechanism called TLAP, and provided 15 security analyses and performance security evaluations. Li et al. [27] proposed a lightweight secure transmission protocol, iTLS, which allows clients to send encrypted data without additional round trips. Compared with the TLS, the iTLS reduces traffic and network handshake delays. Gaba et al. [28] proposed a lightweight mutual authentication scheme called the RLMA, which is used to protect distributed intelligent environments from abuse. The RLMA uses implicit certificates and implements mutual authentication and key negotiations for intelligent devices in the environment, thereby proving the effectiveness of the proposed scheme through formal AVISPA analysis. Li et al. [29] used fuzzy extractors [30] combined with elliptic curve cryptography to develop an identity authentication mechanism for wireless sensor networks. Hammi et al. [31] proposed a one-time password (OTP) identity authentication scheme based on elliptic curve cryptography [32] and demonstrated the security and efficiency of their scheme. Lohachab et al. [33] proposed an elliptic curve-based mechanism for privacy-preserving authentication in distributed networks. The above-mentioned results show that low computing costs, low communication, storage overhead, the realization of identity authentication, and attack resistance are the key issues to achieving IIoT security.

## 3. Problem Definition

**Figure 1:**
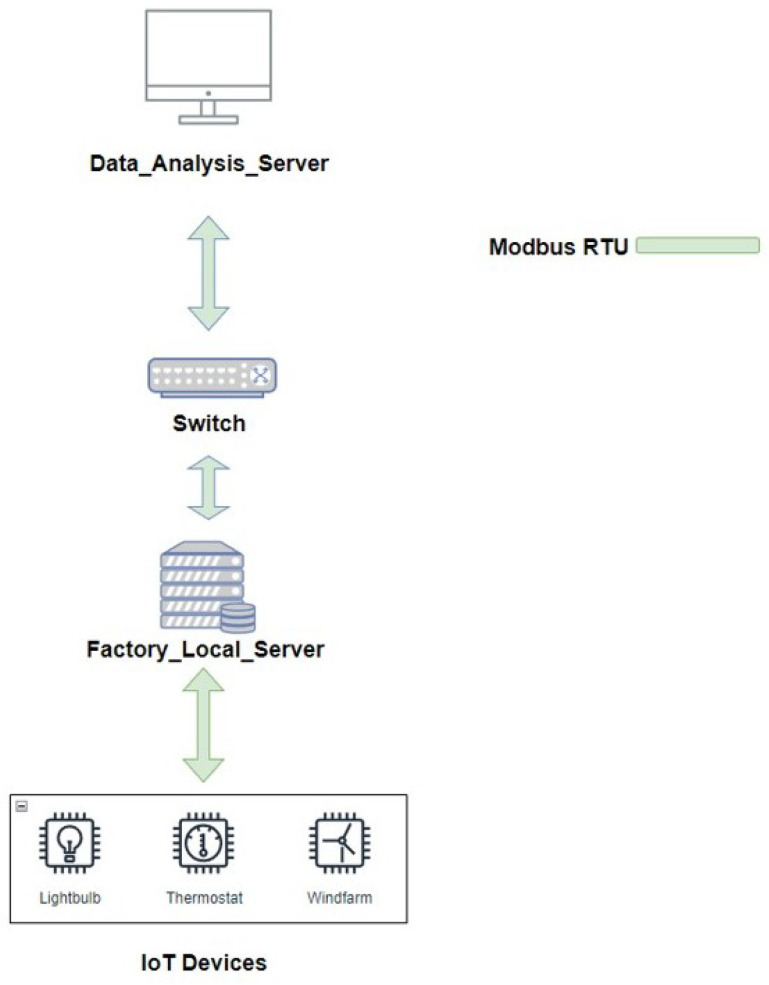
The system without authentication mechanisms in the Industrial Internet of Things.

Figure 1 shows the system architecture without an identity authentication mechanism. In industrial transmission environments without relevant identity authentication mechanisms, devices typically collect data through terminal IoT devices and send them directly to a backend server for subsequent processing or analysis. Data were transmitted using the Modbus protocol.

**Figure 2:**
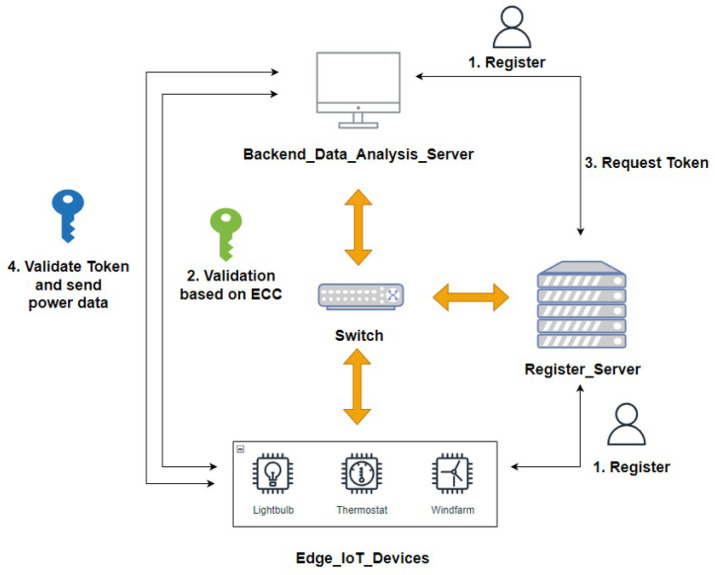
The proposed system.

Figure 2 shows the architecture of the proposed identity authentication system. The identity authentication system proposed in this paper was added to an original industrial environment to establish a secure data transmission environment and protect sensitive data transmitted in the industrial environment. The following is a description of each component, and Table 1 shows the abbreviations.

(1) Register_Server(RS):

The trusted registration server serves two purposes in the experimental scenario presented in this paper:

- To register the identity of terminal devices with the trusted registration server.
- To generate a JWT for devices that pass the first-stage authentication.

In the proposed authentication system, the terminal IoT devices must first request registration from a trusted registration server. The trusted registration server generates a $TID_{i}$ for the terminal IoT device based on the registration request and returns this $TID_{i}$ to both the terminal IoT device and backend data analysis server. When the terminal device identity server and the backend data analysis server pass the first stage of the identity verification mechanism, the trusted registration server generates legitimate tokens required for subsequent data transmission between the two, which are used in the second stage of the authentication mechanism to determine the identity of the legitimate devices and the correctness of the power data.

(2) Backend_Data_Analysis_Server (BDAS):

The backend data analysis server is used to simulate a power company in Taiwan for receiving power data. The server performs two functions.

- After the terminal IoT device has registered its identity, it performs a verification of its legitimacy and generates a session key.
- It verifies the legitimacy of the token transmitted by the terminal IoT device to determine whether the power information in the message originates from a legitimate device.

The trusted registration server returns the $TID_{i}$ of the terminal device to the terminal device identity server. The backend data analysis server performs the first-stage identity authentication using $TID_{i}$ and a terminal device identity server. For legitimate devices that pass the first-stage identity authentication mechanism, the backend data analysis server provides the necessary information to the trusted registration server and applies a set of tokens for legitimate devices that pass the first-stage identity authentication mechanism, which are used for the second-stage identity and data legitimacy verification.

## 4. Process of the Proposed Authentication Mechanism

This study divides the authentication and data transmission processes into four steps:

- The IoT terminal device registers its identity with the registration server and obtains relevant information.
- The first authentication is performed between the IoT terminal device and the backend data analysis server.
- The backend data analysis server obtains a token from the registration server for data transmission.
- The IoT terminal device and the backend data analysis server use the token obtained in the third step for data transmission.

In addition, the authentication process was divided into three stages: initialization, identity registration, and identity authentication.

### 4.1. Initialization Stage and Identity Registration Stage

**Figure 3:**
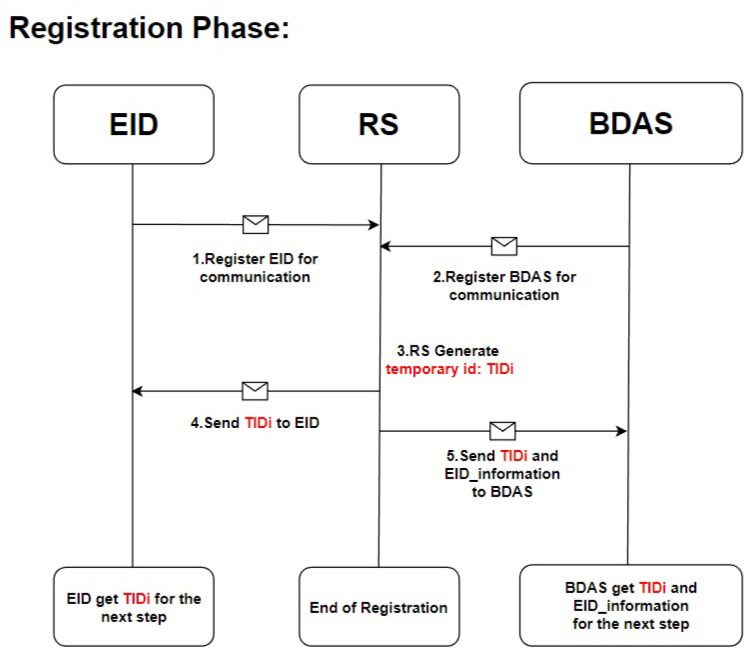
The flow of the registration phase.

In the initialization stage, the registration server publishes the relevant information required for subsequent identity registration. The identity registration stage is divided into five steps, as shown in Figure 3.

- The terminal IoT device sends a request to register its identity with the registration server, and sends the required registration data according to the initialization stage, including EID_ip, EID_port, EID_hostname, and EID_mac_address, to be stored by the registration server.
- The registration server generates a unique temporary ID: $TID_{i}$ for the terminal IoT device, as shown in (1).
- The backend data processing server registers its identity with the registration server by sending BDAS_hostname, BDAS_host_ip, and BDAS_mac_addr.
- The registration server sends $TID_{i}$ to the terminal IoT device.
- The registration server passes $TID_{i}$ and related EID data to the backend data analysis server for the subsequent identity authentication stage.

(1) $$Select\;Temporary\_ID_{i}\left(TID_{i}\right)=Random\;256\;bits$$

### 4.2. Identity Authentication Stage

**Figure 4:**
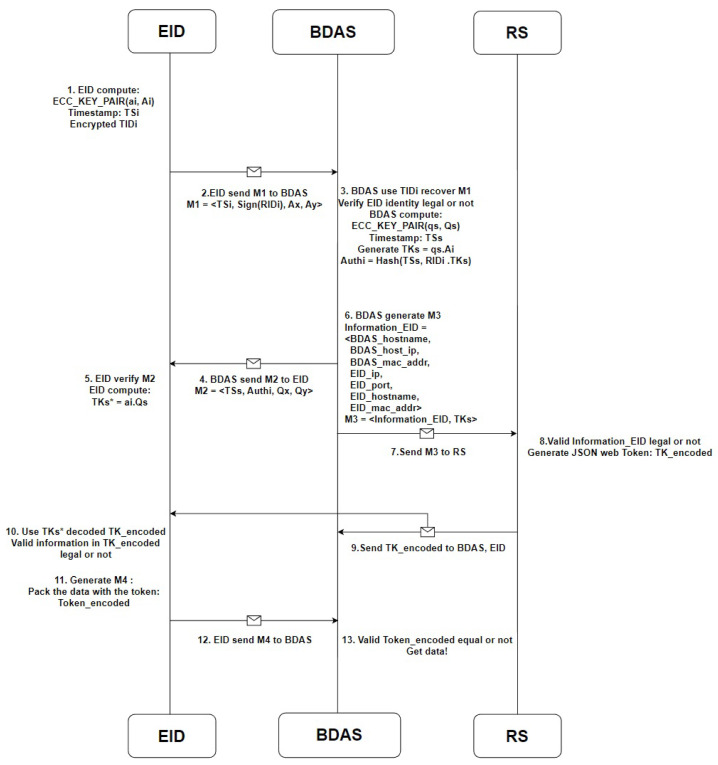
The flow of the identity authentication stage.

In the identity authentication stage, the verification process proposed in this paper is divided into 13 steps, as shown in Figure 4. The verification process is explained from the perspectives of three different devices in the verification mechanism. First, in the local terminal IoT device, which is illustrated by steps 1, 2, 5, 10, 11, and 12 in Figure 4, during the authentication stage, the device first generates an elliptic curve cryptography private key (ai), as shown in (2). According to the generated private key, the device performs elliptic curve–point multiplication to obtain the public key (Ai), as shown in (3). The obtained public key (Ai) is relative to the values of the XY coordinates (Aix and Aiy), as shown in (4). At this stage, the terminal IoT device generates a timestamp ($TS_{i}$) and then uses the $TID_{i}$ obtained in the registration stage to calculate the correct AIDi for verification, as shown in (5). The obtained AIDi is then encrypted, as shown in (6), and the elliptical curve public key (Ai) is XORed with AIDi to obtain the XY-axis values of the public key relative to AIDi, as shown in (7) and (8). Finally, the verification message M1 is sent to the backend data analysis server, as shown in (9). After the transmission of the verification message M1 is completed, the device waits for the backend data analysis server to perform the verification and return the verification message M2. The information contained in the verification message M2 received from the backend data analysis server is shown in (10). The terminal IoT device performs XOR of Qx, Qy, and AIDi in M2 to obtain the elliptic curve cryptography public key of the backend data analysis server, as shown in (11) and (12). After obtaining the public key of the backend data analysis server, the device performs elliptic curve–point multiplication using its own private key to obtain the session key $TK_{S}*$, as shown in (13). The obtained session key is associated with AIDi and a hash function is applied to obtain $Auth_{i}*$, as shown in (14). The device compares $Auth_{i}*$ with $Auth_{i}$. If they are not equal, the connection is terminated. If they are equal, the device waits for a token from the backend data analysis server. After receiving the token, the terminal IoT device uses AIDi to decode the token TK_encoded to obtain relevant information for subsequent data transmission. The token and data are packaged together to form M4, which contains the messages shown in (15).

(2) SelectEID_Private_Key(ai)=Random256bits

(3) EID_Public_Key(Ai)=ai·P

(4) Ai=(Aix,Aiy)

(5) $$Alice\_IDi\left(AIDi\right)=Hash\left(TID_{i}\right)$$

(6) EncryptionAIDi=Sign(AIDi)

(7) Ax=Aix⨁AIDi

(8) Ay=Aiy⨁AIDi

(9) $$M1=<TS_{i},Ax,Ay,Sign\left(AIDi\right)>$$

(10) $$M2=<TS_{s},Qx,Qy,Auth_{i}>$$

(11) Qsx=Qx⨁RIDi

(12) Qsy=Qy⨁RIDi

(13) $$Session\_Key(TK_{s}*)=ai\cdot P$$

(14) $$Authi*=Hash(TK_{s}*\|AIDi)$$

(15) M4=<TK_enocded,Powerdata>

Next, the verification process is explained from the perspective of the backend data analysis server, which is shown by steps 3, 4, 6, 7, and 13 in Figure 4. During the verification phase of the backend data analysis server, the legitimacy of the timestamp in M1 is first checked. If it is not legitimate, the connection is terminated. If it is legitimate, the backend data analysis server computes AIDi by applying a hash function to $TID_{i}$ received during the registration phase, as shown in (5). The backend data analysis server uses a mutually exclusive operation on Ax and Ay in the verification message M1 from the IoT terminal device to obtain the elliptic curve public key (Ai) of the IoT terminal device, as shown in (7) and (8). The backend data analysis server then uses the obtained elliptic curve public key of the IoT terminal device to verify the correctness of the sign (AIDi) in M1, as shown in (16). If it is incorrect, the connection is terminated. If it is correct, the backend data analysis server generates an elliptic curve cryptography private key (qs), as shown in (17), and computes the public key (Qs) using elliptic curve–point multiplication according to the generated private key, as shown in (18). The value of the obtained public key (Ai) relative to the XY coordinates is given by (19). The backend data analysis server then generates a timestamp $TS_{s}$ and uses the obtained elliptic curve public key of the IoT terminal device and the local elliptic curve private key of the backend data analysis server to perform elliptic curve–point multiplication to obtain the session key $TK_{s}$, as shown in (20). After obtaining the session key $TK_{s}$, the backend data analysis server associates it with AIDi and computes the hash function value to obtain the verification message $Auth_{i}$, as shown in (21). The verification message M2 is then packaged and passed to the IoT terminal device for verification; the content of M2 is shown in (10). After M2 is passed, the backend data analysis server generates message M3, the content of which is expressed in (22). The backend data analysis server passes M3 to the registration server for verification and waits for the server to return the token. After receiving the encoded token TK_encoded, the backend data analysis server passes it to the IoT terminal device and waits for the device to return the message M4.

(16) ValidationAIDi=decrypt(Sign(AIDi))

(17) SelectBDAS_Private_Key(qs)=Random256bits

(18) BDAS_Public_Key(Qs)=qs·P

(19) Qs=(Qsx,Qsy)

(20) $$Session\_Key(TK_{s})=qs\cdot Ai$$

(21) $$Auth=Hash\left(TK_{s}\|AIDi\right)$$

(22) M3=<information_EIS,TKs>

After receiving M4, the backend data analysis server compares the token in M4 with that received from the registration server. If they are not the same, the connections are disconnected. If they are the same, the data in M4 are received. Finally, from the perspective of the registration server, the verification process of steps 8 and 9 in Figure 4 is observed. When the registration server receives the message M3 from the backend data analysis server, it first verifies whether the information_EID in M3 is legal. If it is illegal, the token application is rejected. If it is legal, the registration server generates the token TK_encoded, and the token generation method is shown in (23).

(23) TK_encoded=jwt.encode()

## 5. Experiment

### 5.1. Environment Setup

**Figure 5:**
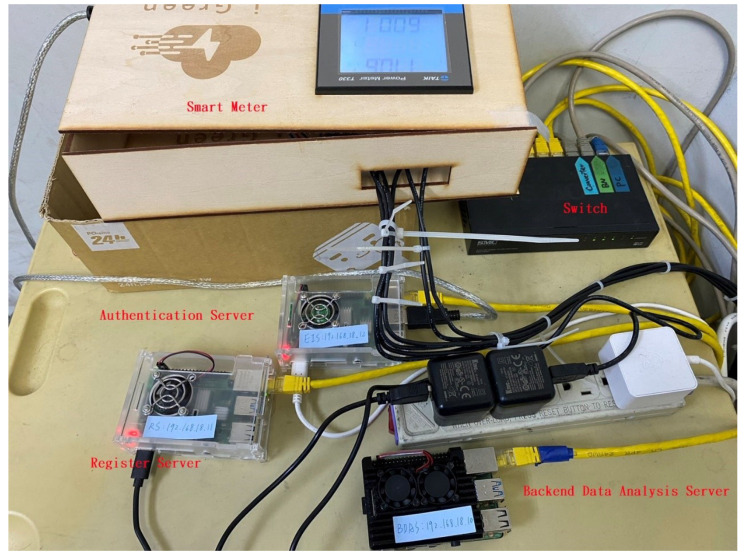
The simulated devices for the proposed method.

This study used a Raspberry Pi4 as the server for building the terminal IoT devices, registration server, and backend data analysis server experimentally, as shown in Table 2. Figure 5 shows a situational diagram of the simulated real-world environment constructed in the experiments conducted in this study. In this study, a Raspberry Pi was used as the server, and Figure 5 shows the smart meter used as the IoT device at the terminal and the server used for implementing the authentication mechanism. The Raspberry Pi used for the terminal device identity server was placed in the meter box, whereas the other servers were the trusted registration server and backend data analysis server. The trusted registration server is used to register the identities of legal devices, whereas the backend data analysis server performs identity authentication and receives the data sent from the terminal IoT devices.

### 5.2. Authentication Phase Based on Elliptic Curve Cryptography

**Figure 6:**
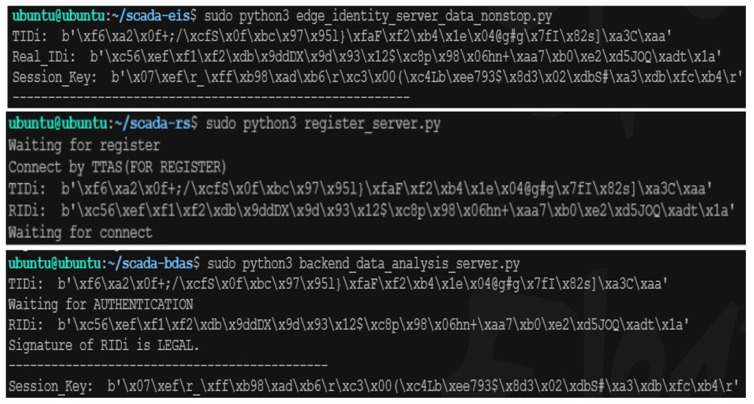
The process of elliptic curve cryptography.

This paper proposes a verification system based on elliptic curve cryptography and the JWT, aiming to prevent the vulnerability of single elliptic curve cryptography or JWT verification mechanism in the face of potential future attacks. Figure 6 illustrates the experimental process of the proposed verification system during the registration phase and the first execution of the authentication mechanism. $TID_{i}$ in Figure 6 is generated and sent by the trusted registration server to the terminal IoT device and backend data analysis server, whereas AIDi is generated locally on each server. According to the first step in Figure 6, both the terminal IoT device and the backend data analysis server generate their own elliptic curve public and private keys. The terminal IoT device encrypts AIDi using its public key. As shown in Figure 6, the backend data analysis server decodes and verifies the correctness of AIDi locally, and both the terminal IoT device and the backend data analysis server generate session keys locally.

### 5.3. Authentication Phase Based on Trusted Tokens

**Figure 7:**
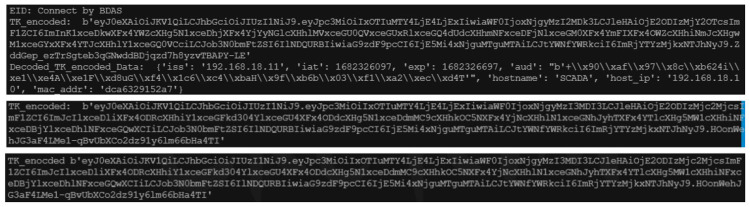
The process of trusted tokens.

Figure 7 shows the experimental process of the second authentication mechanism from the perspectives of the IoT device, trusted registration server, and backend data analysis server. First, the backend data analysis server requests a token from the trusted registration server and passes it to an IoT device. The IoT device then decodes and verifies the content of the token based on the session key it receives and the RIDi. It requests data from the device before packaging it and returns it to the backend data analysis server. Upon receiving this package, the backend data analysis server first compares the tokens to check whether they have been modified. The server accepts the power information sent to the package only if the token has not been modified.

### 5.4. Packet Encryption

**Figure 8:**
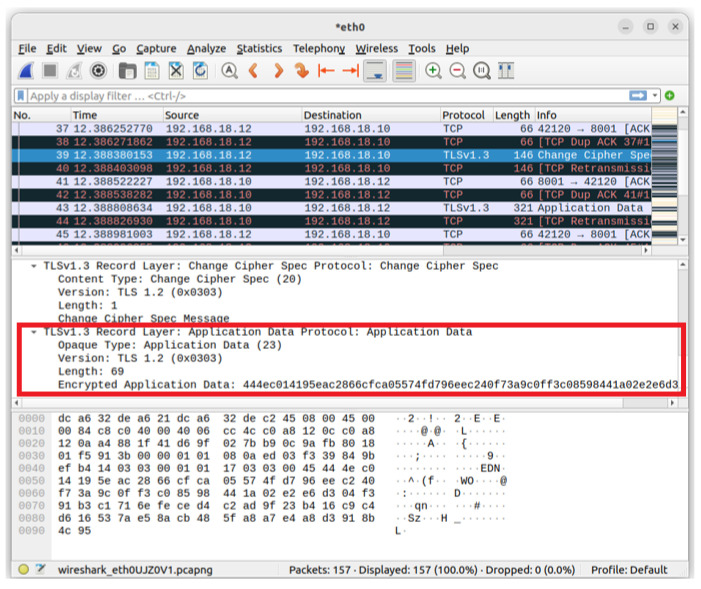
The information of packet encryption.

Figure 8 shows the communication packets between the terminal IoT device and the backend data analysis server during identity verification in the authentication mechanism of this study. The transmitted packets are all encrypted using the TLSv1.3 protocol, making it impossible for attackers to know their contents when intercepting them.

### 5.5. Security Analysis

This section first explains common network attack methods and how the authentication system proposed in this paper can prevent these attacks.

1. **Replay attack:**
  The authentication system proposed in this paper can prevent replay attacks because the identity authentication between the terminal IoT device and the backend data analysis server includes the verification of the timestamps (TSi, TSs) of both parties. When the timestamp exceeds the expiration period, the system determines that the verification request originates from an illegal device and disconnects the connection. Therefore, even if the attacker replays past messages, it will be unable to send data to the terminal IoT device and backend data analysis server owing to the expiration of the timestamp.
2. **Eavesdropping attack:**
  In this study, the TLS protocol is used to encrypt the transmitted packets in the data transmission environment. Therefore, from the attacker’s perspective, the attacker cannot know the data inside the packet based on the stolen packet.
3. **Man-in-the-middle attack:**
  In this study, the TLS protocol is used for packet encryption in data transmission; hence, when an attacker steals the packet, they cannot know the true data transmitted according to the content of the packet. Therefore, the attacker cannot tamper with the data based on the content of the packet. If the user wants to change the encrypted information, namely the token, both the terminal IoT device and the backend data analysis server will check its legitimacy upon receiving it. Therefore, even if the attacker arbitrarily changes the encrypted information, the system will judge it as an illegal message.
4. **Simulated attack:**
  If the attacker wants to send data to the backend data analysis server through a simulated terminal IoT device, the attacker must obtain the AIDi of the terminal IoT device. However, all AIDi are generated locally on each server and, hence, the attacker cannot obtain the value of AIDi. Even if the attacker can skip the first stage of verification and directly enter the second stage of verification, the attacker cannot obtain the session key and, hence, cannot obtain the token used in the second stage of verification. Moreover, when the backend data analysis server receives the message transmitted by the terminal IoT device, it will first check the legitimacy of the token. Therefore, the attacker cannot directly send data to the backend data analysis server.
5. **Support for mutual authentication:**
  The definition of mutual authentication is that both parties authenticate each other in the identity authentication protocol. In the first stage of verification, this article uses AIDi, $Auth_{i}$, and Authi∗ for identity authentication between the terminal IoT device and the backend data analysis server. In the second stage of verification, the token is used for identity authentication between the terminal IoT device and the backend data analysis server.
6. **Support for forward secrecy:**
  The definition of forward secrecy is that the leakage of the long-term main key will not lead to the leakage of past session keys. Forward secrecy can protect past communication from the threat of future key exposure. In each verification process herein, a new session key is generated to encrypt the data. Even if the attacker steals the session key of one communication session, it will not affect the confidentiality of other communications in the future.

### 5.6. Performance Evaluation

**Figure 9:**
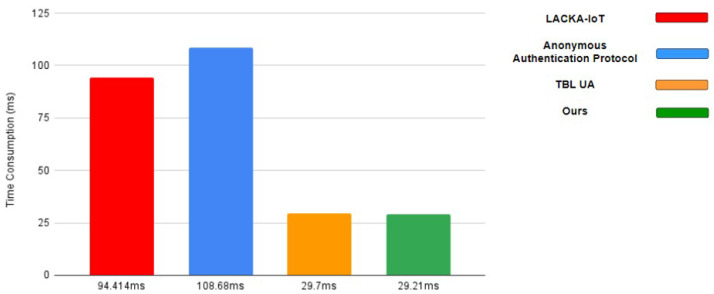
The comparison of time consumption.

This section presents a comparison of the computational costs of this study and related studies. Table 3 lists the time costs and notation descriptions of elliptic curve cryptography, token generation, and other encryption algorithms used in the authentication mechanism. In terms of performance evaluation, we compared the proposed scheme with those presented in several other studies, as shown in Table 4. The authentication mechanisms used in [25,29] were implemented using elliptic curve cryptography, whereas [22] used tokens to achieve identity authentication. The overall time costs of the authentication system were compared with those in [22,25,29]. The identity authentication protocol proposed in [22] is called the TBLUA, whereas that proposed in [25] is called the LACKA-IoT. The study in [29] proposed an anonymous identity authentication protocol, which is referred to as the anonymous authentication protocol in this paper. LACKA-IOT consists of four stages: system setup stage, device registration stage, device access control stage, and dynamic device addition stage. It is noteworthy that LACKA-IOT extensively utilizes elliptic curve point addition combined with elliptic curve–point multiplication in the verification process. In the first step of verification between different devices, the legitimacy of timestamps is checked. Additionally, a registration identity process is designed for new device registration during the dynamic device addition stage. This paper [29] focuses on proposing a secure authentication protocol based on wireless sensor networks. It uses a fuzzy extractor to transform biometric feature information into a fixed-length string, and in their authentication system, they implement the verification process using the fuzzy extractor combined with elliptic curve cryptography. TBLUA authentication consists of four stages: offline smart device and gateway registration stage, user reservation stage, smart-device token allocation stage, and the final login and authentication stage. In the offline smart device and gateway registration stage, the registering authority selects a unique ID for the smart device and generates a 1024-bit random number as the gateway’s ID. In the user reservation stage, users need to register their identity with the registering authority in order to use the functionalities of smart devices. In the smart-device token allocation stage, the gateway periodically distributes user tokens to a group of smart devices. In the login–authentication stage, once the registration process is completed, users can log into the system and perform mutual authentication between users, gateways, and smart devices. After authentication, a session key is established between the user and the smart device, enabling the usage of smart device functionalities. In the user reservation stage, token allocation stage, and login and authentication stage, the message exchange encryption between devices or between devices and users is achieved using exclusive OR operations and hash functions, significantly reducing computational burdens and time costs. The overall verification time of [25,29] is much longer than that of [22] and the authentication system proposed in this paper. The average verification time of 50 verifications was considered as the overall time cost of the authentication system in this study. The time required from the terminal IoT device requesting registration from the registration server to the data being returned to the backend data analysis server after passing through the mutual authentication mechanism was 29.21 ms, which was the fastest among the compared authentication systems, as shown in Figure 9.

## 6. Conclusions

In recent years, with the rapid development of IoT technology, security issues in IIoT environments have become increasingly apparent. Thus, this paper proposes an identity authentication mechanism based on elliptic curve cryptography and tokens to ensure the security of data transmission in IIoT environments. The mechanism aims to to prevent attackers from arbitrarily tampering with or impersonating IoT devices and transmitting abnormal information that may cause data errors. In IoT devices with relatively limited computing resources, elliptic curve cryptography can perform the necessary authentication operations more efficiently, and tokens have high privacy and security, which can prevent the exposure of related privacy information. The combination of elliptic curve cryptography and tokens handles the issues of computing resources and data privacy. Furthermore, because the authentication mechanism proposed in this paper combines elliptic curve cryptography with token-based methods, even after the first stage of elliptic curve cryptography verification, EID and BDAS will still confirm each other’s identities based on tokens. The tokens will be reissued after the expiration of their validity period, ensuring that expired tokens are not exploited by malicious individuals. Therefore, compared to identity authentication mechanisms based solely on elliptic curve cryptography or solely on token-based methods, this paper provides mutual authentication, offering enhanced protection for overall identity verification. In this study, we adopted the TLS protocol for communication. This protocol can encrypt packets during the communication process, thereby protecting the confidentiality and integrity of data, and effectively preventing attackers from tampering with and forging the data. Thus, the security of the entire communication process can be ensured, further enhancing the security of IIoT environments. In terms of security analysis, the proposed mechanism in this paper effectively withstands replay attacks, eavesdropping attacks, man-in-the-middle attacks, and simulated attacks. Additionally, the mechanism supports mutual authentication and forward secrecy. In the experimental results, the proposed mechanism demonstrates approximately 73% improvement in efficiency through the lightweight characteristics of elliptic curve cryptography. Moreover, in the analysis of time complexity, the proposed mechanism exhibits significant effectiveness. In future work, we hope to simplify the authentication process on terminal devices to better adapt them to devices with further limited resources. In addition, the scope envisioned in this paper is the energy management system of the Southern Taiwan Green Energy Science City. In the SCADA system of this domain, Modbus is used for packet transmission and the payload is plaintext, which raises security concerns. Therefore, the proposed architecture in this paper is expected to ensure the network security of SCADA in real industrial IoT energy management systems and prevent attackers from conducting attacks.

## Figures and Tables

**Table 1:** The list of abbreviations.

Notations	Description
EID	Abbreviation of Edge_IoT_Device
RS	Abbreviation of Register_Server
BDAS	Abbreviation of Backend_Data_Analysis_Server
$TID_{i}$	Temporary id for *i*th EID
P	Base point of the elliptic curve: ECC_SECP256R1
ai	Private key of EID
Ai	Public key of EID
·	Elliptic curve–point multiplication operation
(Aix,Aiy)	XY-axis coordinates of the EID public key
$Ts_{i}$	Timestamp generated by EID
Hash()	Hash function
⨁	Exclusive OR operation
‖	Concatenation operation
AIDi	Alice_id for *i*th EID
Sign()	Encrypt RIDi with EID public key
(Ax,Ay)	Hide information of EID public key
$M_{i}$	*i*th verification message
qs	Private key of BDAS
Qs	Public key of BDAS
(Qsx,Qsy)	XY-axis coordinates of the BDAS public key
(Qx,Qy)	Hide information of BDAS public key
$TS_{i}$	Timestamp generated by BDAS
$TK_{s}$	Session key generated by BDAS
$Auth_{i}$	Authentication message generated by BDAS
$TK_{S}*$	Session key generated by EID
$Auth_{i}*$	Authentication message generated by EID
information_EID	Information of EID
BDAS_hostname	Hostname of BDAS
BDAS_host_ip	IP address of BDAS
BDAS_mac_addr	Mac address of BDAS
EID_ip	IP address of EID
EID_port	Socket port of EID
EID_hostname	Hostname of EID
EID_mac_addr	Mac address of EID
TK_encoded	Json Web Token for verification

**Table 2:** The specification of simulated devices.

	Server	OS	Storage
EID	Raspberry Pi4	Ubuntu20.04	64 GB
RS	Raspberry Pi4	Ubuntu20.04	64 GB
BDAS	Raspberry Pi4	Ubuntu20.04	64 GB

**Table 3:** A description of the time-related notation.

Notation	Description
$T_{eca}$	Computational cost of elliptic curve addition
$T_{ecm}$	Computational cost of elliptic curve multiplication
$T_{h}$	Computational cost of the hash function
$T_{xor}$	Computational cost of exclusive OR
$T_{jwt\_enc}$	Computational cost of encode JSON Web Token
$T_{jwt\_dec}$	Computational cost of decode JSON Web Token
$T_{enc}$	Cost of one encryption using symmetric cryptography
$T_{dec}$	Cost of one decryption using symmetric cryptography

**Table 4:** The time complexity of each method.

Method	Total Cost
LACKA-IoT [25]	$3T_{eca}+7T_{ecm}+6T_{h}$
Anonymous Authentication Protocol [29]	$6T_{ecm}+19T_{h}$
TBLUA [22]	$42T_{h}+T_{dec}$
Proposed method	$4T_{ecm}+5T_{h}+T_{jwt\_enc}+T_{jwt\_dec}+8T_{xor}$

## Data Availability

Data sharing not applicable.
